# Gender Differences in the Co-Use of Tranquilizers, Sedatives, Sleeping Pills and Alcohol among Spanish Adolescents: A Nationwide Population-Based Study

**DOI:** 10.3390/children11030339

**Published:** 2024-03-13

**Authors:** Pilar Carrasco-Garrido, Isabel Jiménez-Trujillo, Valentín Hernández-Barrera, Lidiane Lima Florencio, Spencer Yeamans, Domingo Palacios-Ceña

**Affiliations:** 1 Department of Medical Specialties and Public Health, Health Sciences Faculty, Universidad Rey Juan Carlos, 28922 Madrid, Spain; pilar.carrasco@urjc.es (P.C.-G.); isabel.jimenez@urjc.es (I.J.-T.); valentin.hernandez@urjc.es (V.H.-B.); spencer.yeamans@urjc.es (S.Y.); 2 Grupo Consolidado de Investigación en Epidemiología del Medicamento de la Universidad Rey Juan Carlos (RESEPMED), 28922 Madrid, Spain; domingo.palacios@urjc.es; 3 Department of Physical Therapy, Occupational Therapy, Rehabilitation and Physical Medicine, Health Sciences Faculty, Universidad Rey Juan Carlos, 28922 Madrid, Spain

**Keywords:** adolescent, female, tranquilizing agents, ethanol, observational study, health surveys

## Abstract

Adolescence is a critical developmental stage for the initiation of substance use worldwide, which is one of the main risk-taking behaviors that may impact adolescents’ physical and mental well-being. The aims of this study were to (1) assess the prevalence of the co-use of tranquilizers, sedatives, and sleeping pills with alcohol (TSSp&AC) by gender in the Spanish adolescent population in 2018 and (2) identify the variables associated with TSSp&AC. An observational cross-sectional study following STROBE guidelines was conducted. We analyzed data from 38,010 adolescents aged 14 to 18 years old (18,579 males and 19,431 females) who participated in ESTUDES (Survey on Drug Use in Secondary Education in Spain) 2018. Female adolescents reported a higher prevalence of TSSp&AC than males (*p* < 0.001). The factors associated with female co-use were being 16–18 years of age (OR 1.65); the consumption of tobacco (OR 1.73), cocaine (OR 1.84), other illicit psychoactive drugs (OR 1.89); and novel illicit psychoactive drugs (OR 1.74); no perceived health risk from the consumption of TSSps (OR 2.45); and the perceived availability of TSSps (OR 2.23) and alcohol (OR 2.09). There are several factors associated with TSSp&AC in Spanish female adolescents with potential implications for healthcare providers.

## 1. Introduction

Adolescence is a critical developmental stage for the initiation of substance use worldwide, including substances such as tobacco, alcohol, marijuana- and cannabis-based products, cocaine, heroin, and psychoactive substances [1]. It also includes the misuse and use of prescribed drugs such as tranquilizers, sedatives, and sleeping pills (TSSps) and the co-use of several legal or illegal substances [2,3,4,5].

Substance use is one of the main risk behaviors during adolescence that can impact their mental and physical well-being [6]. Adolescents’ consumption pattern includes the combination of alcohol and non-medical prescription drugs, marijuana and alcohol, and marijuana and TSSps [3,4,5]. They also combine energy drinks with other substances such as TSSps, cannabis, alcohol, and tobacco, according to previous studies [6,7,8]. There is a concern that the co-use of different substances and the rise observed in adolescent consumption increases the risk of toxicity, overdoses, and addiction [2,9,10,11]. Several aspects can contribute to the co-use pattern among adolescents, and it seems to be accentuated by their perception of low risk and easy availability [2,3].

Worldwide, more than a quarter of young people aged 15–19 years, i.e., an estimated 155 million adolescents, drink alcohol [9]. The prevalence of heavy episodic drinking among adolescents aged 12–18 years ranges between 5% and 20%, with the rate rising as age increases [12]. Among Spanish adolescents, alcohol is the most used psychoactive substance, with a pattern of use mostly concentrated over the weekend. One-third (31.7%) of adolescents also have binge drinking habits [13].

Public health surveillance data from 2016 show that roughly 1.5 million US adolescents reported tranquilizer/sedative use or misuse in the previous year [14]. Adolescents’ consumption of TSSps has increased during the last few decades, and TSSps are currently among the substances with the lowest age of onset [15]. In Spain, previous studies reported that TSSp misuse by adolescents in Spain increased significantly between 2004 and 2014 [16,17].

Moreover, there are gender-related differences in adolescents’ patterns of consumption [3,16]. A greater prevalence of TSSp, stimulant, and alcohol use can be observed for female adolescents when compared with male adolescents, according to their context and geographic area [17,18,19,20]. In Spain, both alcohol and nonmedical TSSp consumption have increased, especially among females, between 2004 and 2014 [16,17,20]. There is also a distinct consumption pattern related to immigration, with lower drug and substance consumption observed for immigrant adolescents (or those with an immigrant background) when compared to native-born adolescents [21,22].

It has been reported that alcohol consumption is a factor associated with TSSp use [16,17,19,20], but to the best of our knowledge, there have been no studies describing the co-use of TSSps and alcohol (TSSp&AC) among adolescents. Due to the negative impact of substance use among adolescents (e.g., associations with unsafe sex, dangerous driving, violence, and premature deaths) [9], it is important to identify the gender prevalence of TSSp&AC among adolescents. The objectives of the present study were to (1) assess the prevalence of TSSp&AC by gender in the Spanish adolescent population (14–18 years) in 2018 and (2) identify sociodemographic features, lifestyle habits, perceived health risks from consumption, and the perceived availability of substances associated with TSSp&AC among adolescents in Spain in 2018.

## 2. Materials and Methods

### 2.1. Ethics Statement

According to Spanish legislation, the approval of the ethics committee was not necessary since a publicly accessible dataset with anonymous data was used [23]. All the surveys analyzed were anonymous and dissociated and contained no recognizable personal information.

### 2.2. Study Design

For this cross-sectional study, data were obtained from the 2018 Survey on Drug Use in Secondary Education in Spain (ESTUDES 2018), which was conducted in Spain from October 2018 to October 2019.

ESTUDES is an ongoing national survey, performed biannually since 1994 on adolescents between 14 and 18 years old [24]. ESTUDES uses a similar methodology to studies developed in Europe and the USA, which allows for international comparison. The surveys use a two-stage cluster sampling, in which the educational centers were first randomly selected (first-stage units) and then the classrooms (second-stage units). Details of the methodology can be found elsewhere [24]. Briefly, ESTUDES 2018 includes a representative sample of the Spanish adolescent population aged from 14 to 18 years old enrolled in the 3rd and 4th year of Compulsory Secondary Education (ESO), the 1st and 2nd year of Baccalaureate, and the 1st and 2nd year of Basic Vocational Training Cycles and Intermediate Vocational Training Cycles. Excluded from this framework were certain groups such as 14-year-old students in Primary Education, 18-year-old students enrolled in university studies, students between 14 and 18 years of age who did not attend class on the day and time the survey was completed (absentees), students in General Regime Education included in Social Guarantee and Distance Education Programs, and students in Evening and Special Regime Education. Two classrooms per center were chosen for participation in the survey from the same teaching stratum, and the questionnaire was provided to all the students present. All schools and classrooms had the same probability of participating in the survey within each stratum, regardless of their size. Data collection was performed using an anonymous and standardized self-reported questionnaire [24]. For the present study, the sample was stratified by gender, and the results are displayed and compared between males and females. The 2018 survey included 38,010 adolescents of both sexes, aged from 14 to 18.

### 2.3. Study Variables

In ESTUDES 2018, the information used for creating the dependent variables (considered to be dichotomous variables) was obtained from “yes” or “no” answers to the following questions: “Have you taken a tranquilizer, sedative, and/or sleeping pill (TSSp) without a prescription during the last 12 months?” The questionnaire included the following drugs: hypnotics, Trankimazin, Rohypnol, Tranxilium, Diazepam, Valium, barbiturates, Lexatin, Orfidal, Noctamid, benzodiazepines, Zolpidem, etc., but it did not include Valerian, Passiflora, and Dormidina (doxylamine). Respondents were also asked “Have you drunk alcohol during the last 12 months?” and TSSp&AC was determined when adolescents answered “yes” to both questions.

The independent variables were the primary sociodemographic characteristics of the adolescent population—namely age, sex, nationality (Spanish or immigrant), parents’ occupational status (unemployed, employed, or inactive), parents’ educational level (no education, primary school, secondary school, and higher education), perceived family economic situation and population of the town. To determine the use of other substances, we used responses for smoking and energy drink consumption over the previous 12 months (dichotomous variable, yes/no). Also, to determine the use of illegal psychoactive substances, there were questions on the consumption of marijuana, cocaine, heroin, other illicit psychoactive drugs (LSD, non-LSD, hallucinogenic, and amphetamine), and novel psychoactive substances (Ketamine, Spice, Mephedrone, and Ayahuasca) during the previous 12 months.

We also used variables associated with the perceived risk of alcohol and TSSp consumption. For the perceived risk variable, subjects were asked to give their opinions about the health effects and other problems that could result from alcohol and TSSp use. This variable was categorized into some/many problems or none/few problems (no or few problems/quite a few or many problems). The perceived availability of the substances was categorized as “impossible”, “very difficult/easy” or “very easy”.

### 2.4. Statistical Analysis

The prevalence of total TSSp&AC for the ESTUDES 2018 survey was calculated according to the study variables. Data analysis from female and male adolescents was performed. For descriptive statistics, means and standard deviations were calculated for quantitative variables, and proportions for qualitative variables. Student’s *t*-test distribution or the Fisher exact test was used to compare, respectively, means and proportions, and Pearson’s χ^2^ test was used for the bivariate comparison of proportions. Statistical significance was set at *p* < 0.05 (2-tailed).

Multivariate logistic regression analysis was performed to estimate the independent effect of each study variable on TSSp&AC and to obtain the corresponding adjusted odds ratio (aOR) [25]. The variables included in the multivariate analysis were those variables with a significant association in the bivariate analysis and those variables considered relevant in the scientific literature. Once the model was constructed, we analyzed TSSp&AC during the study period and calculated the adjusted odds ratios (aORs). The logistic regression models were constructed in accordance with the following steps: (1) Bivariate analysis of each individual variable was performed; (2) all significant variables were included in the bivariate; (3) the Wald statistic for each variable was used to determine its contribution to the multivariate model; (4) the likelihood ratio test was used to compare the new model with the previous model; and (5) once the final model was constructed, we checked for any linearity and interactions in the model. No significant interactions were found. The aOR with a 95% confidence interval (95% CI) was the measure of association provided by the multivariable models.

Estimates were made by incorporating STATA/SE 16 (STATA Corp, College Station, Texas, TX, USA) sampling weights and using the “svy” (survey command) functions, which enabled us to incorporate the sampling design into all our statistical calculations (descriptive, χ^2^ test, logistic regression). Statistical significance was set at α < 0.05 (2-tailed). The effective response rate in ESTUDES 2018 was 97% [24].

## 3. Results

A total of 38,010 adolescents aged from 14 to 18 or older (48.87% males and 51.12% females) participated in ESTUDES 2018. The study population comprised 2392 females (12.31%) and 1396 males (7.52%), of whom 1111 (6.82%) were aged 14–15 years old and 2677 (12.32%) were aged 16–18 years old. The sociodemographic characteristics of the sample by gender are detailed in Table 1. Females presented a greater prevalence of TSSp&AC than males, with a proportion of 12.31% and 7.52%, respectively (*p* < 0.001). For both genders, TSSp&AC was most prevalent among those aged 16–18 years old, with once again a higher proportion of girls than boys within this age range. Regarding social aspects, the majority of the adolescents’ parents were employed and received secondary or higher education, and most of the adolescents lived in urban environments and reported average perceived incomes. In relation to substance use, consumption was lower among females than males, except for tobacco. Females also more frequently perceived a health-related risk from alcohol or TSSp consumption and considered themselves less informed about drugs than boys. However, females also more frequently felt that both substances were easy or very easy to obtain.

The results of the prevalence of TSSp&AC among male and female adolescents according to sociodemographic variables are presented in Table 2. Compared to males, females presented a greater prevalence of TSSp&AC in the 16–18 age range (OR 1.80), were predominantly native-born (OR 1.73), and had one or both parents employed (OR 1.82 and 1.68, respectively).

The prevalence and association of TSSp&AC according to substance consumption and information, perceived risk, and availability are shown in Table 3. Girls were associated with a higher prevalence of TSSp&AC than boys when both reported consumption of tobacco (OR 1.51), energy drinks (OR 1.91), marijuana (OR 1.63), cocaine (OR 1.65), heroin (OR 2.06), other psychoactive drugs (OR 1.41), and novel psychoactive substances (OR 1.17). The prevalence of girls who reported TSSp&AC was also higher than boys among those who perceived no/few problems related to health risks from alcohol (OR 1.94) or TSSp (OR 2.29) consumption and among those who perceived alcohol (OR 1.74) or TSSp (OR 1.75) availability as “easy” or “very easy”.

The results of the multivariate analysis are shown in Table 4. In our sample, the female gender tended to be a predictor of TSSp&AC (OR, 2.09; 95% CI, 1.82–2.39). When the consumption pattern among female adolescents was analyzed, the variables that were independently and significantly associated with a probability of TSSp&AC were age between 16 and 18 years old (OR, 1.65; 95% CI, 1.39–1.95), smoking (OR, 1.73; 95% CI, 1.42–2.12), energy drinks (OR, 1.4; 95% CI, 1.17–1.66), marijuana (OR, 1.33; 95% CI, 1.08–1.64), cocaine (OR, 1.84; 95% CI, 1.14–2.99), other and novel psychoactive substance consumption (OR, 1.89; 95% CI, 1.33–2.68 and OR, 1.74; 95% CI, 1.08–2.79, respectively) in the previous 12 months. The absence of a perceived health risk for TSSps (OR, 2.45; 95% CI, 1.98–3.03) and the easy availability of alcohol (OR, 2.09; 95% CI, 1.24–3.52) or TSSps (OR, 2–23; 95% CI, 2–65) were also significant associated factors.

## 4. Discussion

Our results showed a 10% prevalence of TSSp&AC during the past 12 months, even with a high rate of the perceived risks of using TSSps or alcohol. Our findings also highlight the factors associated with TSSp&AC when considering female and male adolescents separately. The recognition of the associated factors and users’ main characteristics would enhance the quality of the proposed surveillance and public health strategies.

Our first major finding is that TSSp&AC is more prevalent among girls. Being female was also identified as an important associated factor in TSSp&AC, with a prevalence twice as high for this co-use compared with males. The greater prevalence of TSSp&AC among females is the same pattern observed for the adolescent consumption of TSSps only [16,26]. However, among the studies considering AC only, there is no consistent gender pattern regarding adolescent consumption [27,28]. It is also recognized that gender indicators of AC and related harms have been converging when reviewing data from samples of high school or college students worldwide [29]. A fall in the male–female ratio of any alcohol use from 2.2 to 1.1 was observed when those born in the earliest years of the 20th century are contrasted to those born at the end of the 20th century. In 5% of the studies, the sex ratio was lower than 1, meaning that females have exceeded males in their drinking levels. Most such cases came from cohorts born after 1981.

Potential contributing factors for this gender association could be related to social and epidemiological aspects. Concerning TSSps, they are indicated for affective and sleeping disorders, which are more prevalent among adolescent girls [30,31]. Moreover, the increase in AC among girls could be a generational factor, as current adolescents tend to live with a more egalitarian role model [17], which is reinforced by peers [32,33]. It can reflect the current sociopolitical context since Spanish girls interviewed by Martínez-Manrique et al. [34] associated alcohol consumption with empowerment and the conquest of traditionally masculine spaces. There could also be an influence of the increased targeting of alcohol marketing to young female consumers. Brands are reflecting and reproducing important aspects of feminine identities and women’s day-to-day lives and using spaces and examples that could reflect gender equality to promote alcohol use and encourage consumers [35,36].

Another interesting aspect is that, compared to males, adolescent female immigrants present lower TSSp&AC than native-born adolescents. This agrees with the lower prevalence of risk behaviors among immigrants or those with an immigrant background [37]. It could be partially explained by contextual factors such as lower levels of income or substance use, religion, or culture from their country of origin [37]. However, this lower consumption level among immigrants may only be confirmed for alcohol, not for benzodiazepine or other substances [22,38], and it also occurred where/when adolescents and their families were not yet socially integrated [22,37].

The factors significantly associated with self-reported TSSp&AC in both male and female adolescents were being 16–18 years old, the use of other substances, the perceived availability of TSSp and alcohol (easy/very easy to obtain), and the lack of perceived risk from TSSp use. For female adolescents, the greatest association was observed for those who did not perceive any health risk from TSSp consumption (OR 2.45), while for male adolescents, the greatest association was with other illicit psychoactive drug use in the previous 12 months (OR 2.58). Therefore, the factors associated with this low-risk perception need to be verified. As both TSSps and alcohol are legal substances, their use tends to be more socially accepted, and the underlying risks of their co-use may be underestimated.

However, TSSp&AC is also related to the use of other legal and illicit substances. Moreover, there may be short- and long-term repercussions, such as association with unsafe sex or dangerous driving, violence, and premature death; higher levels of intensive TSSp use in later life [15]; the exacerbation of the severity and development of concurrent mental disorders; and substance use disorder in adulthood [9].

The recognition of the pattern of associated factors within female and male adolescents, with a clear acknowledgment of the differences between them, would help in addressing this increasing health problem properly. Based on our findings, intervention for male adolescents should address the risks of the interaction of polysubstance use (legal and illegal). For female adolescents, emphasis on the real risks of TSSp&AC should be considered. Moreover, in relation to TSSps, patients should be informed of the risks of sharing their prescribed medications and storing them inadequately at home [38]. In addition to the distinct associated factors identified for males and females in our study, sex and gender should always be considered for interventions. For example, when the intervention is directed to the family environment, it should be considered that, for boys, the presence of family conflict is a risk factor associated with alcohol and/or cannabis consumption, while for girls, communication and the presence of consequences for breaking rules reduce the probability of being an alcohol and cannabis user [39].

There are internal and external aspects that cannot be assessed during a nationwide survey but, according to the literature, should be considered to screen potential users and delineate specific interventions. Personal traits such as anxiety sensitivity and impulsivity are specifically associated with sedative/tranquilizer misuse, while impulsivity and sensation seeking are related to alcohol consumption [40,41]. Adolescents with greater empathy and emotional clarity may be most at risk of consuming harmful substances, maybe because consumption tends to occur in the context of leisure and socialization. Conversely, greater emotional repair seems to enhance the possibility of having a healthy life without alcohol and tobacco consumption [42].

Even though an “information paradox” has been identified among adolescents in Spain, i.e., those who perceived themselves as better informed are also those with higher alcohol consumption, there is still a need to offer better-targeted prevention strategies, including participative models [32]. Delivering the information itself may not be enough for prevention. A recent study involving adolescents from Tarragona (Spain) demonstrated that increased polydrug use was associated with unmonitored sources, while greater reliance on supervised sources for information was related to reduced single-substance consumption and polydrug use [43].

According to the second edition of the *International Standards on Drug Use Prevention* [44], the most effective strategies to reduce alcohol use involve evidenced-based interventions such as prevention education based on social competence and influence and parenting skill programs (starting in early adolescence), and community-based multi-component initiatives. In this second edition, there was no evidence specifically demonstrated for the non-medical use of prescription drugs. However, they suggest that the emerging evidence indicating that universal, evidence-based interventions in schools, families, and communities are effective in reducing substance use might apply to the non-medical use of prescription drugs as well.

Based on Spanish adolescents’ perspectives, the interventions for the prevention of alcohol consumption could start before consumption begins at a younger age and include personalized content such as risk reduction and gender-specific training, and they should be adapted to their methods of communication [34]. A web-based computer-tailored intervention has been demonstrated to reduce heavy episodic alcohol consumption among Spanish adolescents [45].

This study has limitations. First, the survey is based on the self-report of substance consumption, and some replies may be socially conditioned. Nevertheless, even though individuals may overestimate or underestimate, surveys are extremely useful for investigating patterns, frequencies, and longitudinal trends of TSSp and alcohol consumption. Second, the study design did not enable us to establish causality owing to the lack of a longitudinal follow-up. Nevertheless, our study provides additional insights into demographic aspects, the perceived health risk of consumption, and perceived TSSp&AC in female adolescents, for whom there is little information at the population level, particularly in Spain.

Future epidemiological studies could focus on the analysis of the TSSp&AC trends over several years since consumption patterns may change over time, while future clinical studies could focus on the risks of the TSSp&AC and on the efficacy of tailored interventions designed along with adolescents to reach them using similar ways to communicate.

## 5. Conclusions

TSSp&AC prevalence was 10% among adolescents between 14 and 18 years old who participated in ESTUDES 2018 in Spain. There was a higher prevalence of TSSp&AC among adolescent females than among males. Females were twice as likely to report TSSp&AC as males. The identified factors associated with greater consumption among females were being 16–18 years old; the use of tobacco, cocaine, and novel illicit psychoactive drugs; the perceived availability of TSSps and alcohol; and the perceived lack of risk from the use of TSSps. Our results have clear implications for health services in Spain, which should develop joint programs focused on the prevention of TSSp&AC and monitoring TSSp and alcohol consumption in female adolescents aged 16–18 years old.

## Figures and Tables

**Table 1:** Characteristics of the study population according to gender; Survey on Drug Use in Secondary Education in Spain (ESTUDES) 2018.

		Male		Female		Total		
		N	%	N	%	N	%	*p*-Value
Age	14–15 years	8005	43.09%	8279	42.61%	16,285	42.84%	0.348
	16–18 years	10,574	56.91%	11,151	57.39%	21,725	57.16%	
	Total	18,579	100.00%	19,431	100.00%	38,010	100.00%	
Nationality	Spanish	16,792	90.65%	17,424	89.87%	34,216	90.25%	0.110
	Immigrants	1733	9.35%	1964	10.13%	3697	9.75%	
Occupational status of parents	Unemployed both	551	3.03%	671	3.50%	1223	3.27%	0.026
	Employed one	6228	34.26%	6616	34.53%	12,844	34.40%	
	Employed both	11,398	62.70%	11,873	61.97%	23,272	62.33%	
Educational level of parents	No formal education	317	1.93%	327	1.82%	644	1.87%	0.000
	Primary school	744	4.52%	936	5.21%	1680	4.88%	
	Secondary school	7154	43.48%	8166	45.52%	15,320	44.55%	
	Higher education	8237	50.07%	8511	47.44%	16,747	48.70%	
Perceived family income	Above average	3012	16.42%	2071	10.80%	5083	13.55%	0.000
	Average	14,519	79.18%	16,249	84.71%	30,768	82.01%	
	Below average	807	4.40%	861	4.49%	1668	4.45%	
Living environment	Rural (<10,000 inhabitants)	2634	14.18%	3074	15.82%	5708	15.02%	0.000
	Urban (≥10,000 inhabitants)	15,945	85.82%	16,357	84.18%	32,302	84.98%	
Any cigarette smoking in the past 12 months	No	12,758	68.67%	11,973	61.62%	24,731	65.06%	0.000
	Yes	5821	31.33%	7458	38.38%	13,279	34.94%	
Energy drink use in the past 12 months	No	9436	50.79%	13,420	69.07%	22,857	60.13%	0.000
	Yes	9143	49.21%	6010	30.93%	15,153	39.87%	
Marihuana use in the past 12 months	No	13,712	73.80%	14,957	76.98%	28,670	75.43%	0.000
	Yes	4867	26.20%	4473	23.02%	9340	24.57%	
Cocaine use in the last 12 months	No	17,986	96.81%	19,113	98.36%	37,099	97.60%	0.000
	Yes	593	3.19%	318	1.64%	911	2.40%	
Heroin use in the last 12 months	No	18,456	99.33%	19,382	99.75%	37,838	99.55%	0.000
	Yes	124	0.67%	48	0.25%	172	0.45%	
Other illicit psychoactive drug use in the last 12 months	No	17,672	95.12%	18,898	97.26%	36,570	96.21%	0.000
	Yes	908	4.88%	532	2.74%	1440	3.79%	
Novel psychoactive substances in the last 12 months	No	18,071	97.26%	19,067	98.13%	37,138	97.70%	0.000
	Yes	508	2.74%	364	1.87%	872	2.30%	
Perceived health risks for consumption of alcohol	No/few problems	4034	26.77%	3554	21.00%	7588	23.72%	0.000
	Quite a few/many problems	11,035	73.23%	13,368	79.00%	24,402	76.28%	
Perceived health risks for consumption of TSSps	No/few problems	1366	10.28%	1257	8.39%	2623	9.28%	0.000
	Quite a few/many problems	11,920	89.72%	13,713	91.61%	25,633	90.72%	
Perceived availability of alcohol	Impossible/very difficult to obtain	859	5.56%	819	4.75%	1678	5.13%	0.000
	Easy/very easy to obtain	14,584	94.44%	16,448	95.25%	31,032	94.87%	
Perceived availability of TSSps	Impossible/very difficult to obtain	4864	52.73%	5196	52.43%	10,060	52.58%	0.001
	Easy/very easy to obtain	4360	47.27%	4714	47.57%	9074	47.42%	
Sufficiently informed about drugs	Yes, perfectly	5744	31.99%	3885	20.41%	9629	26.03%	0.678
	Yes, sufficiently	7322	40.77%	7798	40.96%	15,121	40.87%	
	Only halfway	3619	20.15%	5988	31.46%	9607	25.97%	0.000
	No, I am misinformed	1273	7.09%	1365	7.17%	2639	7.13%	
TSSp co-use with alcohol in the last 12 months	No	17,183	92.48%	17,039	87.69%	34,222	90.03%	0.000
	Yes	1396	7.52%	2392	12.31%	3788	9.97%	

**Table 2:** Prevalence of the co-use of tranquilizers, sedatives, and sleeping pills with alcohol among male and female adolescents according to sociodemographic variables.

		Male			Female			Both			OR Female
Variable	Categories	N	%	*p*-Value	N	%	*p*-Value	N	%	*p*-Value	
Age group	14–15 years	427	5.33	0.000	684	8.26	0.000	1111	6.82	0.000	1.60 (1.37–1-87)
	16–18 years	970	9.17		1708	15.31		2677	12.32		1.80 (1.60–2.00)
	Total	1396	7.52		2392	12.31		3788	9.97		1.72 (1.58–1.89)
Nationality	Spanish	1268	7.55	0.616	2156	12.38	0.293	3424	10.01	0.348	1.73 (1.57–1.90)
	Immigrants	125	7.24		227	11.54		352	9.53		1.67 (1.24–2.26)
Occupational status of parents	Unemployed both	56	10.15	0.042	96	14.23	0.253	151	12.39	0.019	1.47 (0.94–2.31)
	Employed one	450	7.22		820	12.39		1270	9.88		1.82 (1.55–2.13)
	Employed both	865	7.59		1443	12.15		2308	9.92		1.68 (1.50–1.89)
Educational level of parents	No formal education	24	7.6	0.471	46	14.08	0.000	70	10.89	0.000	1.99 (0.96–4.11)
	Primary school	64	8.59		129	13.75		193	11.46		1.69 (1.10–2.60)
	Secondary school	574	8.03		1100	13.47		1674	10.93		1.78 (1.55–2.05)
	Higher education	614	7.45		970	11.4		1584	9.46		1.59 (1.40–1.82)
Perceived family income	Above average	287	9.53	0.000	282	13.61	0.008	569	11.19	0.000	1.49 (1.20–1.86)
	Average	1018	7.01		1962	12.08		2980	9.69		1.82 (1.64–2.02)
	Below average	79	9.79		129	14.99		208	12.47		1.62 (1.09–2.41)
Living environment	Rural (<10,000 inhabitants)	178	6.75	0.109	387	12.58	0.608	565	9.89	0.850	1.98 (1.57–2.53)
	Urban (≥10,000 inhabitants)	1219	7.64		2005	12.26		3223	9.98		1.69 (1.53–1.86)

**Table 3:** Prevalence of the co-use of tranquilizers, sedatives, and sleeping pills with alcohol among male and female adolescents according to substance consumption and information, perceived risk, and availability.

		Male			Female			Both			OR Female
Variable	Categories	N	%	*p*-Value	N	%	*p*-Value	N	%	*p*-Value	
Any cigarette smoking in the past 12 months	Yes	810	13.91	0.000	1462	19.6	0.000	2272	17.11	0.000	1.51 (1.33–1.70)
Energy drink use in the past 12 months	Yes	943	10.31	0.000	1084	18.04	0.000	2027	13.38	0.000	1.91 (1.69–2.17)
Marihuana use in the past 12 months	Yes	760	15.61	0.000	1039	23.23	0.000	1799	19.26	0.000	1.63 (1.43–1.87)
Cocaine use in the last 12 months	Yes	194	32.75	0.000	141	44.52	0.000	336	36.86	0.000	1.65 (1.14–2.38)
Heroin use in the last 12 months	Yes	49	39.34	0.000	28	57.2	0.000	76	44.37	0.000	2.06 (0.84–5.05)
Other illicit psychoactive drug use in the last 12 months	Yes	300	33.08	0.000	219	41.13	0.000	519	36.06	0.000	1.41 (1.06–1.87)
Novel psychoactive substances in the last 12 months	Yes	181	35.51	0.000	143	39.17	0.000	323	37.04	0.000	1.17 (0.82–1.67)
Perceived health risks for consumption of alcohol	No/few problems	348	8.63	0.031	552	15.53	0.000	900	11.86	0.031	1.94 (1.62–2.33)
	Quite a few/many problems	834	7.56		1618	12.11		2453	10.05		1.68 (1.50–1.89)
Perceived health risk for consumption of TSSps	No/few problems	184	13.46	0.000	330	26.22	0.000	513	19.57	0.000	2.29 (1.76–2.97)
	Quite a few/many problems	852	7.15		1580	11.52		2432	9.49		1.69 (1.51–1.89)
Perceived availability of alcohol	Impossible/very difficult to obtain	29	3.38	0.000	44	5.39	0.000	73	4.36	0.000	1.63 (0.90–2.93)
	Easy/very easy to obtain	1211	8.3		2235	13.59		3446	11.1		1.74 (1.58–1.91)
Perceived availability of TSSps	Impossible/very difficult to obtain	291	5.97	0.000	478	9.2	0.000	768	7.64	0.000	1.60 (1.30–1.94)
	Easy/very easy to obtain	654	15.01		1114	23.64		1769	19.49		1.75 (1.53–2.01)
Sufficiently informed about drugs	Yes, perfectly	530	9.22	0.000	592	15.23	0.000	1121	11.65	0.000	1.77 (1.50–2.08)
	Yes, sufficiently	505	6.9		954	12.23		1459	9.65		1.88 (1.62–2.18)
	Only halfway	227	6.27	0.000	665	11.1	0.000	892	9.28	0.000	1.87 (1.53–2.28)
	No, I am misinformed	71	5.58		120	8.79		191	7.24		1.63 (1.13–2.35)

TSSps: tranquilizers, sedatives, and sleeping pills.

**Table 4:** Results of the multivariable regression models showing those variables significantly and independently associated with self-reported data on the co-use of tranquilizers, sedatives, and sleeping pills with alcohol among male and female adolescents.

		Male	Female	BOTH
Age	16–18 Years	1.26 (1–1.58)	1.65 (1.39–1.95)	1.49 (1.3–1.7)
Any cigarette smoking in the past 12 months	Yes	1.61 (1.21–2.14)	1.73 (1.42–2.12)	1.68 (1.42–1.98)
Energy drink use in the past 12 months	Yes	1.45 (1.15–1.82)	1.4 (1.17–1.66)	1.41 (1.23–1.61)
Marihuana use in the past 12 months	Yes	1.67 (1.24–2.26)	1.33 (1.08–1.64)	1.44 (1.21–1.7)
Cocaine use in the last 12 months	Yes	1.54 (1.03–2.3)	1.84 (1.14–2.99)	1.74 (1.27–2.37)
Other illicit psychoactive drug use in the last 12 months	Yes	2.58 (1.86–3.58)	1.89 (1.33–2.68)	2.19 (1.72–2.8)
Novel psychoactive substances in the last 12 months	Yes	1.52 (1–2.31)	1.74 (1.08–2.79)	1.56 (1.14–2.14)
Perceived health risk for consumption of TSSps	No/few problems	1.52 (1.14–2.03)	2.45 (1.98–3.03)	2.05 (1.73–2.44)
Perceived availability of alcohol	Easy/very easy	NS	2.09 (1.24–3.52)	1.93 (1.32–2.82)
Perceived availability of TSSps	Easy/very easy	2.04 (1.63–2.55)	2.23 (1.88–2.65)	2.12 (1.85–2.44)
Sex	Female	NA	NA	2.09 (1.82–2.39)

TSSps: tranquilizers, sedatives, and sleeping pills. NS: not significant. NA: not applicable.

## Data Availability

The data presented in this study are available in this article.
